# The Association of Cytomegalovirus and Allostatic Load by Country of Birth and Length of Time in the United States

**DOI:** 10.3390/diseases11030101

**Published:** 2023-08-04

**Authors:** Matthew Hill, Sayed Mostafa, Perpetua M. Muganda, Liesl K. Jeffers-Francis, Emmanuel Obeng-Gyasi

**Affiliations:** 1 Department of Built Environment, North Carolina A&T State University, Greensboro, NC 27411, USA; 2 Environmental Health and Disease Laboratory, North Carolina A&T State University, Greensboro, NC 27411, USA; 3 Department of Mathematics and Statistics, North Carolina A&T State University, Greensboro, NC 27411, USA; 4 Department of Biology, North Carolina A&T State University, Greensboro, NC 27411, USA

**Keywords:** cytomegalovirus, allostatic load, stress, country of birth, length of time in US

## Abstract

Background: Cytomegalovirus (CMV) is a highly prevalent virus with a worldwide distribution. It typically remains dormant in most individuals until reactivation. Immunocompromised states are known to be potential causes for CMV reactivation. Current research has shown a link in the decline of immigrant health among those living in the US for an extended period, though the impact of CMV on this is not clear. Methods: This study investigated the association between country of birth, duration of US residency, allostatic load, and latent cytomegalovirus infection (CMV IgG) in a sample of US adults aged 20–49. The data utilized for our analysis was obtained from the National Health and Nutrition Examination Survey (NHANES) conducted between 2001 and 2004. Allostatic load, an index measuring the cumulative physiological strain on the body as it strives to regain stability in the presence of chronic stress, provided a valuable approach to assess stress within the context of CMV exposure. Logistic regression modeling was employed to estimate odds ratios and confidence intervals for the analysis. The chi-square test of association and Cramer’s V statistic were used to assess the correlation among categorical variables, while Pearson’s correlation coefficient was applied to evaluate the relationship between continuous variables. The results revealed that individuals born outside the US and those with less than 20 years of residency in the US exhibited significantly higher proportions of positive CMV IgG compared to individuals born in the US. Specifically, individuals born outside the US had more than triple the odds of CMV IgG when adjusting for the AL index (OR = 3.69, *p*-value = 0.0063). A similar trend was observed when examining AL risk based on the duration of US residency. Furthermore, age and sex were identified as significant predictors (*p*-value < 0.05) of AL risk, considering the individual’s country of birth. In summary, the findings of this study significantly enhance our comprehension of the intricate interplay between cytomegalovirus (CMV) and allostatic load (AL). The investigation sheds light on how CMV and AL interact within specific demographic contexts, providing valuable insights into the underlying risk factors for CMV infection.

## 1. Introduction

### 1.1. Cytomegalovirus Epidemiology and Diagnosis

Cytomegalovirus (CMV) is a widespread herpesvirus infection which is highly prevalent in the US and globally. It spreads through close contact with bodily fluids, including saliva, urine, semen, tears, and breast milk. Transmission can also occur during childbirth, organ transplantation, and blood transfusions. This virus can remain latent for periods and reactivates when additional factors, such as stress, are present [1,2,3]. Due to this temporary latency of the virus, it is mainly non-life threatening to healthy individuals. Pregnant individuals and those with weakened immune systems face increased risks and potentially fatal outcomes from CMV. Factors like age and race may influence CMV seroprevalence [4]. Diagnosis of CMV is primarily handled via laboratory examination, as clinical symptoms are not always present in individuals. Monitoring virus replication and serum immunological analysis are the most common laboratory methods when assessing CMV [5,6,7]. Past and recent exposure to the virus are measured using CMV-specific immunoglobulin M and G, respectively [5,8,9].

### 1.2. Immigrant Health in the United States

The country of origin may affect an individual’s health in the US. The healthy immigrant effect suggests that immigrants initially have better health than native populations but their health may decline over time, possibly due to increased stressors in their new environment [10].

Specifically, studies have indicated that the health of immigrants to the US tends to be better than US-born individuals but this tends to level off after about 10–20 years of living in the US [10]. Once an immigrant enters the later years of their life, they tend to have poorer health compared to the native US population [10]. Ethnicity impacts health outcomes for immigrants in the US, causing additional stress and limited access to necessary care due to disparities [11].

### 1.3. Immigration and Cytomegalovirus

A person’s CMV exposure risk may be impacted by their country of origin [10]. CMV seroprevalence is highest in the Eastern Mediterranean region (90%), while the Western Pacific and African regions have similar rates at 88% [12]. The financial condition of an individual has a major impact on their access to healthcare and their living conditions. People with lower incomes tend to have higher CMV prevalence worldwide [11]. These stressors impact the individual’s immune system. Children born to individuals from high CMV prevalence developing countries have lower CMV prevalence in their new nation [10].

### 1.4. CMV Serostatus Association with Socioeconomic Factors

In the US, a multivariate model controlling for various factors found an age-adjusted association between family income categories and CMV seroprevalence [13]. In subsequent studies, Dowd et al. found that education and family income, after adjusting for race/ethnicity, were strongly associated with infection status. Each additional year of schooling in the family reduced the likelihood of infection by 5%. Increasing household income from $15,000 to $40,000 was linked to a 14% lower risk of infection [11].

Race and ethnicity were also a factor when examining CMV seroprevalence. When considering family socioeconomic status (SES), non-Hispanic Black participants had a 37% higher risk of CMV exposure as compared to non-Hispanic White participants. Mexican American participants had a 93% higher chance of contracting CMV than non-Hispanic White individuals. In fully adjusted models, smoking increased CMV seropositivity in females but lowered it in males [11]. Finally, household crowding was associated with a higher likelihood of CMV infection [14,15].

### 1.5. Allostatic Load

Allostatic load (AL) is defined as the cumulative burden on a person’s body to try to reestablish allostasis due to chronic stress [16]. Allostasis is the process in which the body attempts to regain homeostasis [17]. This stress can come from lifestyle choices such as drinking and smoking as well as biological factors [18]. As this stress builds up, it can cause allostatic load when an individual is unable to adapt, which has been shown to weaken the immune system and potentially cause adverse health outcomes [11] repeatedly. It is essential to consider how this stress impacts a person as this may be involved in disease processes [19,20,21].

Standard practice has involved the combination of metabolic, immunological, and cardiovascular biomarkers to calculate an AL score [19,22].

### 1.6. Study Objectives

This study aims to examine the relationship between stress and immigration, particularly the role of CMV, by investigating the impact of country of birth and length of time in the US on AL and CMV IgG. Using data from NHANES 2001–2004, we analyzed sociodemographic factors associated with these variables.

**Figure 1:**
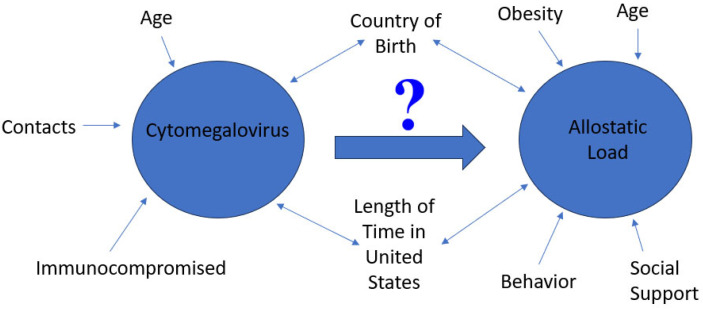
A figure showing the central objective of this study, which is to assess the role of cytomegalovirus on allostatic load by country of birth and length of time in the United States. Critical factors related to CMV and AL are noted in the figure.

This study is vital in understanding the link between immigration, AL and its impact on persistent CMV infection. CMV infection continues to be endemic worldwide; thus, gaining a better understanding of the factors associated with its elevation are critical for population health. The country of birth and length time in the US are critical variables in assessing the distribution and determinants of CMV exposure. These, coupled with the chronic burden of stress, demonstrated by AL, are vital in assessing the health outcomes of at-risk populations. The central objective of this study is found in Figure 1.

## 2. Materials and Methods

### 2.1. Study Population

The study population consisted of US adults age 20–49 years. The sample was a combination of the NHANES 2001–2002 and 2003–2004 participants. The analysis sample consisted of individuals who possessed the necessary data for CMV IgG, which included 4545 participants. We also subset this data to study those with the necessary biomarkers and sociodemographic factors for the AL index (1119 participants). The NHANES survey uses a stratified multistage cluster sample of non-institutionalized US persons. The survey data also incorporates sampling weights and primary sampling units’ information that can be used to obtain weighted estimates of US population parameters.

### 2.2. AL Index

For this study, we constructed the AL index by combining biomarkers from the metabolic, cardiovascular, and immune systems. Previous studies have found this method of AL scoring to be informative in evaluating the cumulative stress of an individual [16,19,23,24,25]. The AL scoring assessment included the measurement of several biomarkers. These biomarkers encompassed a range of health indicators, such as high-density lipoprotein (HDL) cholesterol, total cholesterol (TC), triglycerides (TG), glycosylated hemoglobin (HBA1C), C-reactive protein (CRP), albumin (Alb), systolic blood pressure (SBP), diastolic blood pressure (DBP), body mass index (BMI), and creatinine clearance (CLCR). Next, we divided these biomarkers into survey-weighted quartiles in the database. AL markers, Alb, CLCR, and HDL used the bottom 25% for high-risk consideration. All other biomarkers used the top 25% for high-risk consideration. High-risk and low-risk participants were assigned a 1 and 0, respectively, for each biomarker for a cumulative score out of 10. For our study we considered an AL value of 3 or above to be high based on the literature [16,19,22,26].

### 2.3. Measurement of Study Variables

Demographic variables in this study were obtained through the NHANES computer-assisted personal interview software program. These include age, sex, race/ethnicity, country of birth, length of time in the US, income, physical activity, alcohol use, education, and tobacco consumption. Measurement of critical markers for the AL index and cytomegalovirus have been reported elsewhere [27].

### 2.4. Description of Study Variables and Covariates

The outcome variables of focus for this study include AL and CMV IgG. Covariates included in the study are age, sex (Male/Female), race/ethnicity, education, annual family income, alcohol consumption, current tobacco use, physical activity, country of birth, and length of time in the US. Annual family income was initially measured with the following intervals, [$0–$4999], [$5000–$9999], [$10,000–$14,999], [$15,000–$19,999], [$20,000–$24,999], [$25,000–$34,999], [$35,000–$44,999], [$45,000–$54,999], [$55,000–$64,999], [$65,000–$74,999], and [$75,000 and over]. The responders who wished to not select a specific interval for this variable chose from the following two options, [Over $20,000] and [Under $20,000]. This variable was then converted to the poverty binary variable based on income statistics for the years 2001–2004. The study examined the educational attainment of the participants, encompassing a diverse range of levels. The education variables were categorized as follows: individuals who had completed college and earned a degree or achieved education beyond the college level; those with some college education or an Associate of Arts (AA) degree; high school graduates with a General Educational Development (GED) certification or equivalent qualification; individuals who completed 9th to 11th grade (including 12th grade without obtaining a diploma); and those with educational attainment below the 9th-grade level. Race/Ethnicity was categorized as non-Hispanic (NH) White people, NH Black people, Mexican American, Other Hispanic, and Other/Multiracial. Physical activity responses included yes, no, and unable to do. The assessment of current smoking status involved labeling individuals as “yes” if they had smoked at least one cigarette in the past month. As for alcohol consumption, individuals were categorized as “yes” if they consumed at least 12 alcoholic drinks in a year, and otherwise labeled as “no.” Country of birth was labeled either Born in the US, Mexico, or elsewhere. Time in the US was measured with the following intervals, [Less than 1 year], [1- less than 5 years], [5- less than 10 years], [10- less than 15 years], [15- less than 20 years], [20- less than 30 years], [30- less than 40 years], [40- less than 50 years], and [50 or more years]. Due to multicollinearity, this variable was collapsed to be binary.

### 2.5. Statistical Analysis

All analyses for this study were executed using R programming language version 4.1.1 [28] and accounted for the sampling design and weights of the NHANES dataset. Initially, summary statistics for AL, country of birth, length of time in the US, and CMV IgG were analyzed for patterns in the data. The chi-square test and Cramer’s V statistic were used to measure associations between categorical variables while Pearson correlations were used for continuous variables. Logistic regression models were used to estimate odds ratios and their corresponding 95% confidence intervals. A *p*-value < 0.05 was considered significant in all our analyses.

## 3. Results

### 3.1. Summary Statistics of Key Study Variables

Table 1 shows summary statistics by CMV IgG status and includes both continuous and categorical variables used in this study. For the continuous variables, survey weighted means and standard errors (SE) of the sample mean are shown in parentheses. For categorical variables, survey weighted proportions are shown. The first line of the table shows survey weighted totals with the corresponding survey weighted proportion in parentheses. *p*-Values were obtained using Wilcoxon rank-sum test for complex survey samples and chi-squared test with Rao and Scott’s second-order correction. The total (unweighted) number of individuals with the data necessary for CMV IgG was 4545. This equates to 124,348,343 (weighted) participants as shown in the table. The overall column shows proportions for each variable which can be multiplied by the total weighted number of participants to obtain the weighted totals for each variable. We also note that the number of unknowns for each variable is not shown in the table, but still accounts for the missing proportions.

The average age of participants was 34.81 years for CMV IgG. Most women were positive for CMV IgG (60.08%) while only (47.99%) of men were positive for CMV IgG. We also found that most of the study consisted of NH White participants (67.6%). Most NH White people were negative for CMV IgG (57.85%) compared to every other race/ethnicity which were all predominantly positive for CMV IgG. Individuals born outside the US were primarily positive for CMV IgG with proportions of 92.17% and 80.10% compared to their negative counterparts 7.83% and 19.90%, respectively. Of the participants with an education less than ninth grade, more than twice as many were born outside the US compared to in the US. Individuals with this education level were also predominantly positive for CMV IgG while college graduates were mainly negative. Most people living in the US for less than 20 years were positive for CMV IgG (86.05%) whereas those living in the US for at least 20 years showed an almost 50/50 split in CMV seropositivity. A similar relationship was found when it came to the poverty line. Those making at or below the poverty line were more likely to be positive for CMV IgG (65.42%) compared to those above the poverty line (50.49%).

Table 2 contains summary statistics by AL as well as survey weighted proportions, means, and standard errors of the sample mean in parentheses. This table also shows survey weighted proportions in parentheses on the first line. *p*-Values were obtained using Wilcoxon rank-sum test for complex survey samples and chi-squared test with Rao and Scott’s second-order correction. The total (unweighted) number of participants was 1119 which equates to a total of 29,114,886 (weighted) number of participants. Examining these, we found that on average high AL individuals (37.35) tend to be slightly older as opposed to low AL (32.18). Of the individuals who spent less than 20 years in the US, more than five times as many were positive for CMV IgG compared to negative. NH White people and NH Black people made up the majority of those who spent more than 20 years in the US. Among racial groups, other Hispanic individuals are majority high AL. Lastly, we see a 50/50 split in terms of AL when looking at those who lived in the US for 20+ years. This trend flips for those who lived in the US for less than 20 years, as nearly twice as many are low AL compared to high AL.

### 3.2. Correlation of CMV, AL, Biomarkers and Sociodemographic Factors

**Figure 2:**
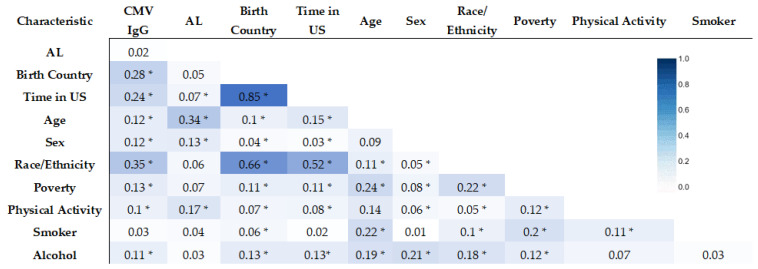
Correlation matrix of AL Risk, CMV IgG, and sociodemographic factors (Cramer’s V statistic) (* statistically significant association, *p*-value < 0.05).

To assess the relationship between our variables of interest, correlation matrices were developed using the Pearson correlation coefficient for continuous variables and the Cramer’s V statistic for categorical variables. In Figure 2, we see that Birth Country and Time in the US had the strongest association (0.85) based on Cramer’s V statistic. We also found Birth Country and Time in the US to be significantly associated with Race/Ethnicity (0.66 and 0.52, respectively).

**Figure 3:**
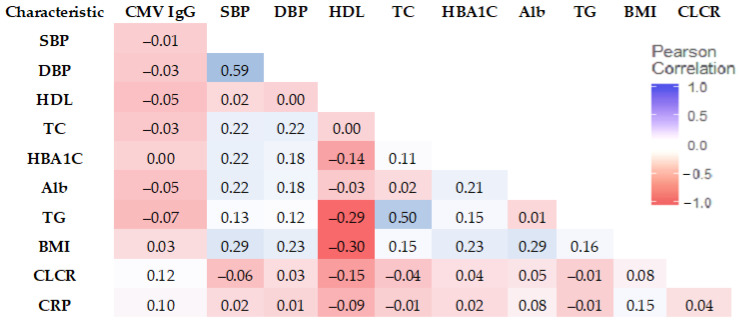
Correlation matrix of CMV IgG and biomarkers (Pearson correlation coefficient).

We next assessed the relationship between biomarkers, CMV IgG, and AL Risk with the correlation matrix. SBP showed the strongest correlation with DBP (0.59). TG and TC had a correlation of 0.50 (See Figure 3).

### 3.3. Logistic Regression Modeling

To obtain odds ratios and identify potential risk factors, we developed logistic regression models for CMV IgG status as well as AL status. These models accounted for potential confounding factors by adjusting for age, sex, race, tobacco smoking, and alcohol consumption where possible. Due to the high collinearity between birth country and time in the US, we subset the sample into two groups for modeling: those born outside the US and those born in the US.

Our initial model of CMV IgG focuses on those born in the US and, due to multicollinearity, we did not adjust for length of time in the US and race in any of our models for CMV IgG. We also did not adjust for poverty in our overall model due to multicollinearity. Akaike information criterion (AIC), odds ratios, confidence intervals, and *p*-values from these models are reported in Table 3. AIC shows how well a model fits the data it is generated from and can be used to compare which model fits the data better. Sex was found to be a significant predictor of the CMV IgG status (*p*-value = 0.0456) among those born in the US. On average, being male cuts the odds of positive CMV IgG (0.49) in half. This was the only significant predictor at the 5% level for modeling CMV IgG among US born individuals using AL risk. Next, we looked at those born outside the US and our model found alcohol consumption to be the only significant predictor (*p*-value = 0.0350) at the 5% level. Lastly, we examined both groups of US born and non-US born individuals.

Next, we included AL index in the models for the CMV IgG status to assess the association between these two variables. We did not adjust for poverty in these models due to multicollinearity between poverty and birth country. The model AIC, odds ratios, *p*-value, and confidence intervals for this model are found in Table 4. Among those born in the US, sex was again found to be significant (*p*-value = 0.0424) and reduces the risk of CMV IgG (0. 49) by the same amount as in the model without AL index. This was the only significant predictor at the 5% level for this model. For those born outside the US, AL index was found to more than triple the odds of CMV IgG on average (OR = 3.69, *p*-value = 0.0063).

When it comes to modeling AL risk (low vs. high), we found age and sex to be significant at the 5% level. On average, each additional year in age (*p*-values = 0.004 and 0.004) is associated with a 6% increase in the odds of having high AL for our models including birth country and time in US, respectively (Table 5). Looking at sex (*p*-value = 0.008 and 0.005), we see that on average men are more than twice as likely to have high AL compared to women for both models.

## 4. Discussion

### 4.1. Implications of Results

This study found no association between CMV IgG and AL. The results matched the work of our prior study which found no association between CMV IgM and AL [27]. This suggests, though potentially associated with stressors, CMV may not impact chronic physiological stress as manifested in AL. According to research by Reed et al., older stressed people exhibited a greater percentage of aged T cells regardless of CMV control, indicating a stronger correlation between stress and immunological deterioration independent of CMV [29].

In this study, we found that age was a significant factor in the AL risk of an individual. AL, as discussed earlier, represents the cumulative burden of stress on the physiology and health of an individual. Works by Crimmins et al. found that AL increases dramatically between the ages of 20 to 60 [30]. These findings align with prior studies on AL that show older individuals on average have higher AL scores [31,32]. This also agrees with other literature on the association of AL risk and age [30]. Aging leads to a decline in cognitive functioning, which has been shown to be associated with AL [33].

Aside from age, sex was found to be a significant factor impacting the AL risk of a person. Our findings suggest that men are more likely to have higher AL risk. Previous studies have shown that men on average have higher AL than females [34], while a study by Remes et al. [35] found that women were twice as likely to suffer from severe stress and anxiety as men. These differences may be partly explained by the fact that AL may not capture all facets of stress and surveys on stress may not capture the biological response to stress. Nevertheless, AL has generally been found to be higher in men than women [36,37].

Results of this study indicated that a greater proportion of women were positive for CMV IgG with a significant relationship found between CMV IgG and sex for those born in the US. This aligns with the current literature which has found that more women tend to be positive for CMV IgG compared to men [38]. This may be in part due to occupation with day care workers, who tend to be women, having higher CMV infection rates [39,40]. In addition the intersection of genetics [41,42], ethnicity [43], and current health status [44,45] may help to explain this finding. That said, sex differences in CMV are more dramatic early in life but these differences become less with age. Women of childbearing age are at risk of CMV and their exposure risk impacts CMV seroprevalence in women [46]. A study found that one to two percent of pregnant women in Western Europe and the United States have CMV primary infection [47].

Young pregnant women who have already had one or more children are most susceptible to developing primary infections during pregnancy. An initial infection posed a 5.9 percent annual risk to pregnant women in the US who had tested negative for the virus in their prior pregnancy [48].

The risk of primary fetal infection during the first trimester was recently found to be startlingly higher in women who were seronegative during their first pregnancy and then became pregnant again within two years—roughly 19 times higher than the risk in the general population. Additionally, it was discovered that their offspring had a five-fold increased risk of developing associated sequelae [49].

Based on our model, drinking alcohol significantly reduced the odds of non-US-born individuals being positive for CMV IgG. One would logically expect that alcohol consumption would increase the odds of CMV infections since CMV is largely transmitted through bodily fluids and the sharing of drinks may promote its spread. That said, the variable used for alcohol consumption had some limitations in that it examined consuming 12 alcoholic drinks in the year which may not be enough drinks to tag an individual as an alcohol consumer. A case report found a relationship between cytomegalovirus and alcohol in an HIV-negative patient with Pneumocystis carinii pneumonia [50] but the design of their study limits the generalizability of the results.

Among those born outside the US, AL index was found to significantly influence CMV seropositivity. This could potentially be due to the healthy immigrant effect (HIE), which posits that those born outside the US have a health advantage that diminishes overtime. This gives rise to non-US born individuals with greater chances of high AL score and thus a potentially weakened immune system which makes them more susceptible to infections such as CMV. Simply put, higher stress levels have been associated with a weakened immune system [51]. This also explains why AL index was not found to be significant for those born in the US as the HIE does not apply to native-born individuals. The birth country was also found to be significantly associated with CMV IgG. Occupational as well as environmental factors play a role in this disadvantage for non-US born individuals [52].

### 4.2. Limitations

This study has limitations including incomplete data for variables like time in the US and AL risk, resulting in information loss. The alcohol variable used only measures whether individuals consumed at least 12 alcoholic beverages in a year, without differentiating current and non-current drinkers or reflecting chronic alcohol consumption accurately. High multicollinearity between time in the US and birth country limited the variables adjusted for in logistic regression models. Other infectious diseases affecting or affected by AL, such as HIV, hepatitis B and C, and tuberculosis, were not considered. The study did not capture the stress of moving to a different country without support networks, which could impact AL. Factors related to CMV reactivation were not fully explored due to their inclusion in the AL index. The cross-sectional design restricts determining temporality. Nevertheless, this study makes progress in understanding the relationship between CMV, AL, and country of birth.

### 4.3. Strengths

This study’s strengths include the fact that it addressed an important research gap by investigating the association between country of birth, duration of US residency, allostatic load, and latent cytomegalovirus infection (CMV IgG). By exploring the relationship between CMV and allostatic load within specific demographic contexts, the study provides valuable insights into the risk factors for CMV infection.

In addition, this study utilizes data obtained from the National Health and Nutrition Examination Survey (NHANES) which is a nationally representative survey that provides a robust dataset for analysis, enhancing the generalizability of the findings. Overall, these strengths contribute to the scientific knowledge regarding the relationship between CMV, allostatic load, and demographic factors, expanding our understanding of the risk factors and implications of CMV infection.

## 5. Conclusions

Our study adds to the growing research on prevalent diseases in the US population. We did not find a significant association between time in the US and CMV IgG or AL risk. Instead, sociodemographic factors including birth country and sex had significant associations with CMV seropositivity. Country of birth was related to alcohol consumption among those born outside of the US which is critical to note since AL is also related to country of birth.

Our findings suggest a need for educating individuals on stress management as well as alcohol use to lower overall AL.

Future work in this area should seek to gain a better understanding of other potential influencers of the critical variables within this study. It is also important for future studies to consider the interaction between sociodemographic factors by potentially assessing their significance using an index like AL.

## Figures and Tables

**Table 1:** Survey weighted proportions, means, and standard errors of the sample mean by CMV IgG.

		CMV IgG		
Characteristic	Overall, N = 124,348,343	Negative, n = 57,026,831 (45.86%)	Positive, n = 67,321,512 (54.14%)	*p*-Value
Age, years (SE)	34.81 (0.21)	34.31 (0.30)	35.23 (0.25)	0.011
Sex				<0.001
Female	50.84%	39.92%	60.08%	
Male	49.16%	52.01%	47.99%	
Race/Ethnicity				<0.001
NH White people	67.16%	57.85%	42.15%	
Mexican American	9.90%	16.20%	83.80%	
NH Black people	12.41%	21.33%	78.67%	
Other and/or Multi-racial	5.29%	22.49%	77.51%	
Other Hispanic	5.24%	29.95%	70.05%	
Poverty Line ($20,000)				<0.001
Above	74.26%	49.51%	50.49%	
At or Below	23.64%	34.58%	65.42%	
Education Level				<0.001
College graduate or above	24.46%	60.91%	39.09%	
Some college or AA degree	32.72%	47.61%	52.39%	
High school graduate/GED or equivalent	26.43%	43.19%	56.81%	
9–11th grade (includes 12th grade with no diploma)	12.01%	28.72%	71.28%	
Less than 9th grade	4.31%	11.34%	88.66%	
Alcohol Consumption				<0.001
Yes	35.70%	48.55%	51.45%	
No	11.28%	35.49%	64.51%	
Currently Smoking				0.200
Yes	30.22%	44.23%	55.77%	
No	16.82%	47.58%	52.42%	
Muscle Strengthening Activities				<0.001
Yes	32.69%	52.84%	47.16%	
No	66.15%	42.34%	57.66%	
Unable to Do	1.15%	49.88%	50.12%	
Birth Country				<0.001
Born in US	83.62%	51.87%	48.13%	
Born in Mexico	6.42%	7.83%	92.17%	
Born elsewhere (Not US nor Mexico)	9.97%	19.90%	80.10%	
Time in US				<0.001
20 or more years	87.60%	50.37%	49.63%	
Less than 20 years	12.29%	13.95%	86.05%	
SBP average reported to examinee	116.33 (0.33)	116.29 (0.53)	116.36 (0.35)	0.500
DBP average reported to examinee	71.56 (0.27)	71.43 (0.36)	71.67 (0.35)	0.700
HDL-cholesterol (mg/dL)	50.61 (0.30)	50.87 (0.58)	50.40 (0.36)	0.700
Total cholesterol (mg/dL)	196.62 (0.78)	195.89 (1.24)	197.24 (1.00)	0.300
Triglycerides (mg/dL)	141.24 (4.11)	145.15 (6.08)	137.96 (5.32)	0.400
Glycohemoglobin (%)	5.33 (0.01)	5.28 (0.02)	5.36 (0.02)	<0.001
Albumin, urine (ug/mL)	22.47 (2.53)	21.26 (4.15)	23.50 (2.73)	<0.001
Body Mass Index (kg/m^2^)	27.86 (0.12)	27.62 (0.18)	28.07 (0.17)	0.023
Creatinine, urine (mg/dL)	140.46 (2.56)	136.72 (2.93)	143.62 (3.03)	0.045
C-reactive protein(mg/dL)	0.39 (0.01)	0.35 (0.01)	0.43 (0.02)	0.012
AL Index	2.53 (0.08)	2.55 (0.10)	2.52 (0.14)	>0.900
AL Risk				0.600
High	10.92%	42.58%	57.42%	
Low	12.46%	45.02%	54.98%	

**Table 2:** Survey weighted proportions, means, and standard errors of the sample mean by AL.

		AL		
Characteristic	Overall, N = 29,114,886	Low, n = 15,491,141 (53.21%)	High, n = 13,623,745 (46.79%)	*p*-Value
Age (years)	34.60 (0.39)	32.18 (0.53)	37.35 (0.46)	<0.001
Sex				<0.001
Female	48.59%	59.70%	40.30%	
Male	51.41%	47.07%	52.93%	
Race/Ethnicity				0.500
NH White	66.70%	53.22%	46.78%	
Mexican American	9.97%	59.65%	40.35%	
NH Black people	13.28%	51.86%	48.14%	
Other and/or Multi-racial	5.27%	51.79%	48.21%	
Other Hispanic	4.78%	44.87%	55.13%	
Poverty Line ($20,000)				0.200
Above	74.51%	51.36%	48.64%	
At or Below	23.66%	59.75%	40.25%	
Education Level				0.200
College graduate or above	20.33%	52.88%	47.12%	
Some college or AA degree	34.68%	56.40%	43.60%	
High school graduate/GED or equivalent	27.44%	54.76%	45.24%	
9–11th grade (includes 12th grade with no diploma)	12.05%	45.29%	54.71%	
Less than 9th grade	5.21%	43.31%	56.69%	
Alcohol Consumption				0.400
Yes	72.79%	53.81%	46.19%	
No	23.36%	49.73%	50.27%	
Currently Smoking				0.600
Yes	29.22%	51.23%	48.77%	
No	16.65%	47.14%	52.86%	
Muscle Strengthening Activities				0.002
Yes	32.59%	65.48%	34.52%	
No	66.68%	47.41%	52.59%	
Unable to Do	0.73%	34.79%	65.21%	
Birth Country				0.200
Born in US	83.77%	52.06%	47.94%	
Born elsewhere (Not US nor Mexico)	9.24%	58.43%	41.57%	
Born in Mexico	6.99%	60.05%	39.95%	
Time in US				0.029
20 or more years	88.29%	52.01%	47.99%	
Less than 20 years	11.57%	62.99%	37.01%	
SBP average reported to examinee	115.88 (0.51)	111.10 (0.69)	121.31 (0.85)	<0.001
DBP average reported to examinee	72.39 (0.58)	68.63 (0.67)	76.67 (0.44)	<0.001
HDL-cholesterol (mg/dL)	49.79 (0.45)	53.34 (0.47)	45.76 (0.75)	<0.001
Total cholesterol (mg/dL)	194.05 (2.20)	180.42 (2.01)	209.56 (3.18)	<0.001
Triglycerides (mg/dL)	141.23 (4.95)	99.72 (4.41)	188.42 (10.24)	<0.001
Glycohemoglobin (%)	5.33 (0.02)	5.17 (0.02)	5.51 (0.04)	<0.001
Albumin, urine (ug/mL)	34.78 (8.00)	19.28 (5.59)	52.40 (15.51)	0.005
Body Mass Index (kg/m^2^)	27.60 (0.18)	25.12 (0.22)	30.43 (0.39)	<0.001
Creatinine, urine (mg/dL)	147.42 (3.59)	154.95 (4.54)	138.85 (4.09)	0.004
C-reactive protein(mg/dL)	0.35 (0.03)	0.23 (0.03)	0.48 (0.04)	<0.001
CMV IgG				0.600
Negative	43.82%	54.67%	45.33%	
Positive	56.04%	52.20%	47.80%	

**Table 3:** CMV IgG model using AL Risk (Adjusted for Age, Sex, Alcohol, and tobacco consumption).

CMV IgG						
	Born in the US		Born Outside the US		Overall	
	OR (2.5%, 97.5%)	*p*-Values	OR (2.5%, 97.5%)	*p*-Values	OR (2.5%, 97.5%)	*p*-Value
Age	1.02 (0.98, 1.06)	0.1980	1.04 (0.95, 1.13)	0.6330	1.02 (0.99, 1.06)	0.192
Sex						
Male	0.49 (0.24, 0.98)	0.0456	1.80 (0.46, 7.00)	0.3500	0.48 (0.24, 0.94)	0.036
Female	Reference					
Alcohol Consumption						
Yes	0.54 (0.21, 1.43)	0.1848	0.06 (0.01, 0.78)	0.0350	0.46 (0.16, 1.31)	0.125
No	Reference					
Currently Smoking						
Yes	1.33 (0.76, 2.32)	0.2693	2.48 (0.75, 8.21)	0.1190	1.40 (0.84, 2.34)	0.173
No	Reference					
AL Risk						
High	0.91 (0.50, 1.66)	0.7211			1.10 (0.61, 2.00)	0.714
Low	Reference					
Poverty Line						
Above	0.64 (0.33, 1.25)	0.1645	0.68 (0.11, 4.10)	0.6330		
At or Below	Reference					

**Table 4:** CMV IgG model using AL Index (Adjusted for Age, Sex, Alcohol, and tobacco consumption).

CMV IgG						
	Born in the US		Born Outside the US		Overall	
	OR (2.5%, 97.5%)	*p*-Values	OR (2.5%, 97.5%)	*p*-Values	OR (2.5%, 97.5%)	*p*-Value
Age	1.02 (0.99, 1.06)	0.1897	0.99 (0.89, 1.11)	0.8991	1.02 (0.99, 1.06)	0.173
Sex						
Male	0.49 (0.25, 0.97)	0.0424	0.75 (0.07, 8.55)	0.7778	0.49 (0.25, 0.95)	0.038
Female	Reference					
Alcohol Consumption						
Yes	0.45 (0.17, 1.24)	0.1090	0.99 (0.08, 12.09)	0.9887	0.45 (0.16, 1.29)	0.118
No	Reference					
Currently Smoking						
Yes	1.44 (0.85, 2.45)	0.1553	1.84 (0.29, 11.73)	0.4358	1.39 (0.84, 2.32)	0.172
No	Reference					
AL Index	0.96 (0.82, 1.12)	0.5448	3.69 (1.75, 7.76)	0.0063	1.00 (0.85, 1.18)	0.992
Time in the US						
Less than 20 years			0.33 (0.00, 27.34)	0.5505		
20 or more years	Reference					
Birth Country						
Born in Mexico					20.62 (6.71, 63.36)	<0.001
Born elsewhere (Not US nor Mexico)					6.21 (1.54, 25.09)	0.017
Born in the US	Reference					
Model AIC	496.4671		59.3118		600.3614	

**Table 5:** AL models using birth country and time in US (Adjusted for age, sex, Alcohol, and tobacco consumption).

AL				
	Using Birth Country		Using Time in the US	
	OR (2.5%, 97.5%)	*p*-Values	OR (2.5%, 97.5%)	*p*-Value
Age	1.06 (1.03, 1.10)	0.004	1.06 (1.02, 1.10)	0.004
Sex				
Male	2.06 (1.30, 3.29)	0.008	2.11 (1.35, 3.31)	0.005
Female	Reference			
Alcohol Consumption				
Yes	0.45 (0.16, 1.27)	0.110	0.45 (0.16, 1.27)	0.114
No	Reference			
Currently Smoking				
Yes	0.86 (0.38, 1.98)	0.689	0.86 (0.38, 1.94)	0.686
No	Reference			
CMV IgG				
Positive	1.05 (0.55, 2.00)	0.867	1.07 (0.57, 2.01)	0.807
Negative	Reference			
Birth Country				
Born in Mexico	0.96 (0.43, 2.15)	0.913		
Born elsewhere (Not US nor Mexico)	0.87 (0.42, 1.81)	0.673		
Born in US	Reference			
Poverty Line				
Above	0.87 (0.38, 1.98)	0.702	0.84 (0.38, 1.86)	0.631
At or Below	Reference			
Time in US				
Less than 20 years			0.70 (0.29, 1.67)	0.375
20 or more years	Reference			
Model AIC	616.4932		615.3074	

## Data Availability

The NHANES dataset is publicly available online, accessible at cdc.gov/nchs/nhanes/index.htm (accessed on 18 March 2023).

## References

[1] Collins-McMillen D., Rak M., Buehler J.C., Igarashi-Hayes S., Kamil J.P., Moorman N.J., Goodrum F. (2019). Alternative promoters drive human cytomegalovirus reactivation from latency. Proc. Natl. Acad. Sci. USA.

[2] Reeves M., Sinclair J. (2008). Aspects of human cytomegalovirus latency and reactivation. Human Cytomegalovirus.

[3] Sinclair J. (2008). Human cytomegalovirus: Latency and reactivation in the myeloid lineage. J. Clin. Virol..

[4] Fowler K.B., Ross S.A., Shimamura M., Ahmed A., Palmer A.L., Michaels M.G., Bernstein D.I., Sánchez P.J., Feja K.N., Stewart A. (2018). Racial and ethnic differences in the prevalence of congenital cytomegalovirus infection. J. Pediatr..

[5] Jahan M. (2010). Laboratory diagnosis of CMV infection: A review. Bangladesh J. Med. Microbiol..

[6] Razonable R.R., Humar A. (2019). Cytomegalovirus in solid organ transplant recipients—Guidelines of the American Society of Transplantation Infectious Diseases Community of Practice. Clin. Transplant..

[7] Prakash K., Chandorkar A., Saharia K.K. (2021). Utility of CMV-specific immune monitoring for the management of CMV in solid organ transplant recipients: A clinical update. Diagnostics.

[8] Basson J., Tardy J., Aymard M. (1989). Pattern of anti-cytomegalovirus IgM antibodies determined by immunoblotting. Arch. Virol..

[9] Nielsen S.L., Sörensen I., Andersen H.K. (1988). Kinetics of specific immunoglobulins M, E, A, and G in congenital, primary, and secondary cytomegalovirus infection studied by antibody-capture enzyme-linked immunosorbent assay. J. Clin. Microbiol..

[10] Markides K.S., Rote S. (2019). The healthy immigrant effect and aging in the United States and other western countries. Gerontologist.

[11] Dowd J.B., Aiello A.E., Alley D.E. (2009). Socioeconomic disparities in the seroprevalence of cytomegalovirus infection in the US population: NHANES III. Epidemiol. Infect..

[12] Cannon M.J., Schmid D.S., Hyde T.B. (2010). Review of cytomegalovirus seroprevalence and demographic characteristics associated with infection. Rev. Med. Virol..

[13] Staras S.A., Dollard S.C., Radford K.W., Flanders W.D., Pass R.F., Cannon M.J. (2006). Seroprevalence of cytomegalovirus infection in the United States, 1988–1994. Clin. Infect. Dis..

[14] Feinstein L., Douglas C.E., Stebbins R.C., Pawelec G., Simanek A.M., Aiello A.E. (2016). Does cytomegalovirus infection contribute to socioeconomic disparities in all-cause mortality?. Mech. Ageing Dev..

[15] Dowd J.B., Haan M.N., Blythe L., Moore K., Aiello A.E. (2008). Socioeconomic gradients in immune response to latent infection. Am. J. Epidemiol..

[16] Guidi J., Lucente M., Sonino N., Fava G.A. (2021). Allostatic load and its impact on health: A systematic review. Psychother. Psychosom..

[17] Sterling P. (2004). Principles of Allostasis: Optimal Design, Predictive Regulation, Pathophysiology, and Rational Therapeutics.

[18] Siew R.V.K., Nabe-Nielsen K., Turner A.I., Bujtor M., Torres S.J. (2022). The role of combined modifiable lifestyle behaviors in the association between exposure to stressors and allostatic load: A systematic review of observational studies. Psychoneuroendocrinology.

[19] Theall B., Wang H., Kuremsky C.A., Cho E., Hardin K., Robelot L., Marucci J., Mullenix S., Lemoine N., Johannsen N.M. (2020). Allostatic stress load and CMV serostatus impact immune response to maximal exercise in collegiate swimmers. J. Appl. Physiol..

[20] Dai S., Mo Y., Wang Y., Xiang B., Liao Q., Zhou M., Li X., Li Y., Xiong W., Li G. (2020). Chronic stress promotes cancer development. Front. Oncol..

[21] McEwen B.S. (2004). Protection and damage from acute and chronic stress: Allostasis and allostatic overload and relevance to the pathophysiology of psychiatric disorders. Ann. N. Y. Acad. Sci..

[22] Bahreinian S., Ball G.D.C., Leek T.K.V., Colman I., McNeil B.J., Becker A.B., Kozyrskyj A.L. (2013). Allostatic Load Biomarkers and Asthma in Adolescents. Am. J. Respir. Crit. Care Med..

[23] Duong M.T., Bingham B.A., Aldana P.C., Chung S.T., Sumner A.E. (2017). Variation in the Calculation of Allostatic Load Score: 21 Examples from NHANES. J. Racial Ethn. Health Disparities.

[24] Petrovic D., Pivin E., Ponte B., Dhayat N., Pruijm M., Ehret G., Ackermann D., Guessous I., Younes S.E., Pechère-Bertschi A. (2016). Sociodemographic, behavioral and genetic determinants of allostatic load in a Swiss population-based study. Psychoneuroendocrinology.

[25] Rainisch B.K.W., Upchurch D.M. (2013). Sociodemographic Correlates of Allostatic Load Among a National Sample of Adolescents: Findings From the National Health and Nutrition Examination Survey, 1999–2008. J. Adolesc. Health.

[26] Geronimus A.T., Hicken M., Keene D., Bound J. (2006). “Weathering” and age patterns of allostatic load scores among blacks and whites in the United States. Am. J. Public Health.

[27] Hill M., Obeng-Gyasi E. (2022). The Association of Cytomegalovirus IgM and Allostatic Load. Diseases.

[28] R Development Core Team (2022). R: A Language and Environment for Statistical Computing.

[29] Reed R.G., Presnell S.R., Al-Attar A., Lutz C.T., Segerstrom S.C. (2019). Perceived stress, cytomegalovirus titers, and late-differentiated T and NK cells: Between-, within-person associations in a longitudinal study of older adults. Brain Behav. Immun..

[30] Crimmins E.M., Johnston M., Hayward M., Seeman T. (2003). Age differences in allostatic load: An index of physiological dysregulation. Exp. Gerontol..

[31] Kaestner R., Pearson J.A., Keene D., Geronimus A.T. (2009). Stress, Allostatic Load, and Health of Mexican Immigrants. Soc. Sci. Q..

[32] Taylor J., McFarland M.J., Carr D.C. (2019). Age, Perceptions of Mattering, and Allostatic Load. J. Aging Health.

[33] Gruenewald T.L., Seeman T.E., Karlamangla A.S., Sarkisian C.A. (2009). Allostatic load and frailty in older adults. J. Am. Geriatr. Soc..

[34] Juster R.-P., Pruessner J.C., Desrochers A.B., Bourdon O., Durand N., Wan N., Tourjman V., Kouassi E., Lesage A., Lupien S.J. (2016). Sex and gender roles in relation to mental health and allostatic load. Psychosom. Med..

[35] Remes O., Brayne C., Van Der Linde R., Lafortune L. (2016). A systematic review of reviews on the prevalence of anxiety disorders in adult populations. Brain Behav..

[36] Kerr P., Kheloui S., Rossi M., Désilets M., Juster R.-P. (2020). Allostatic load and women’s brain health: A systematic review. Front. Neuroendocrinol..

[37] Obeng-Gyasi E., Obeng-Gyasi B. (2020). Chronic stress and cardiovascular disease among individuals exposed to lead: A pilot study. Diseases.

[38] Hecker M., Qiu D., Marquardt K., Bein G., Hackstein H. (2004). Continuous cytomegalovirus seroconversion in a large group of healthy blood donors. Vox Sang..

[39] Adler S. (1990). Cytomegalovirus and child day care: Evidence for an increased infection rate among day care workers. Pediatr. Infect. Dis. J..

[40] Pass R.F., Hutto C., Lyon M.D., Cloud G. (1990). Increased rate of cytomegalovirus infection among day care center workers. Pediatr. Infect. Dis. J..

[41] Misra M.K., Mishra A., Pandey S.K., Kapoor R., Sharma R.K., Agrawal S. (2015). Genetic variation in micro-RNA genes of host genome affects clinical manifestation of symptomatic human cytomegalovirus infection. Hum. Immunol..

[42] Goldeck D., Larsen L.A., Christiansen L., Christensen K., Hamprecht K., Pawelec G., Derhovanessian E. (2016). Genetic influence on the peripheral blood CD4+ T-cell differentiation status in CMV infection. J. Gerontol. Ser. A Biomed. Sci. Med. Sci..

[43] Duchowny K.A., Noppert G.A. (2021). The Association Between Cytomegalovirus and Disability by Race/Ethnicity and Sex: Results From the Health and Retirement Study. Am. J. Epidemiol..

[44] Zhen J., Zeng M., Zheng X., Qiu H., Cheung B.M.Y., Xu A., Wu J., Li C. (2022). Human cytomegalovirus infection is associated with stroke in women: The US National Health and Nutrition Examination Survey 1999–2004. Postgrad. Med. J..

[45] Al-Attar A., Presnell S.R., Peterson C.A., Thomas D.T., Lutz C.T. (2016). Data correlations between gender, cytomegalovirus infection and T cells, NK cells, and soluble immune mediators in elderly humans. Data Brief.

[46] Hyde T.B., Schmid D.S., Cannon M.J. (2010). Cytomegalovirus seroconversion rates and risk factors: Implications for congenital CMV. Rev. Med. Virol..

[47] Colugnati F.A., Staras S.A., Dollard S.C., Cannon M.J. (2007). Incidence of cytomegalovirus infection among the general population and pregnant women in the United States. BMC Infect. Dis..

[48] Fowler K.B., Stagno S., Pass R.F. (2004). Interval between births and risk of congenital cytomegalovirus infection. Clin. Infect. Dis..

[49] Leruez-Ville M., Guilleminot T., Stirnemann J., Salomon L.J., Spaggiari E., Faure-Bardon V., Magny J.-F., Ville Y. (2020). Quantifying the burden of congenital cytomegalovirus infection with long-term sequelae in subsequent pregnancies of women seronegative at their first pregnancy. Clin. Infect. Dis..

[50] Ikawa H., Hayashi Y., Ohbayashi C., Tankawa H., Itoh H. (2001). Autopsy case of alcoholic hepatitis and cirrhosis treated with corticosteroids and affected by Pneumocystis carinii and cytomegalovirus pneumonia. Pathol. Int..

[51] Glaser R., Kiecolt-Glaser J. (2009). Stress damages immune system and health. Discov. Med..

[52] Barnett E.D., Walker P.F. (2008). Role of Immigrants and Migrants in Emerging Infectious Diseases. Med. Clin. N. Am..

